# Oxytocin as a regulator of skeletal muscle plasticity and metabolic function

**DOI:** 10.3389/fendo.2026.1863901

**Published:** 2026-07-09

**Authors:** Joao da Cruz-Filho, Danilo Lustrino

**Affiliations:** Laboratory of Basic and Behavioral Neuroendocrinology (LANBAC), Department of Physiology, Center for Biological and Health Sciences, Federal University of Sergipe, São Cristóvão, Brazil

**Keywords:** muscle mass, oxytocin, oxytocin receptor, proteolysis, skeletal muscle

## Abstract

Although skeletal muscle has historically been considered a non-target tissue of oxytocin (OXT), accumulating evidence demonstrates that this neuropeptide influences myogenesis, regeneration, and protein metabolism, thereby modulating muscle plasticity through neuroendocrine signaling axes. Among physiological and pathological contexts, variations in circulating OXT are associated with changes in muscle mass. For example, anabolic steroid exposure promotes muscle hypertrophy alongside increased OXT levels, whereas aging and diabetes are characterized by muscle loss and reduced OXT. Mechanistically, OXT activates its receptor (OXTR), a G protein–coupled receptor, engaging Gαq signaling, intracellular calcium mobilization, and downstream pathways such as Akt–FoxO, thus linking central neuropeptide signaling to peripheral metabolic regulation. This crosstalk inhibits proteolysis while stimulating protein synthesis. Consistent with these findings, preclinical and clinical studies support a protective role of OXT in muscle mass regulation. However, key aspects of OXT biology in skeletal muscle remain poorly understood, including the regulation of its synthesis, degradation, and secretion under physiological and pathological conditions, which may influence its local and systemic actions. As a myokine, OXT may integrate local muscle signaling with systemic neuroendocrine actions, operating through autocrine, paracrine and endocrine mechanisms, although the relative contributions remain unresolved. In this context, emerging evidence on OXT signaling in skeletal muscle, particularly its role in regulating muscle plasticity, provides a conceptual framework that is explored throughout this review.

## 1 Introduction

One of the earliest advances in understanding the endocrine activity of the pituitary gland occurred in the late nineteenth century, when George Oliver and Edward A. Schäfer (1895) demonstrated that extracts from this gland, when administered intravenously in dogs, produced a significant increase in blood pressure (1). A few years later, Henry Dale (1906) observed that extracts from the posterior pituitary (neurohypophysis) induced uterine contractions in pregnant cats, indicating the presence of an active principle with uterotonic properties, later called oxytocin (from the Greek *oxys*, rapid, and *tokos*, birth) (2). Subsequently, Isaac Ott and John C. Scott (3),along with Edward A. Schäfer and Kenneth Mackenzie (4), demonstrated that these same pituitary extracts also induced milk ejection from the mammary gland, indicating that oxytocin (OXT) was likewise responsible for this physiological effect. However, it was not until 1953 that Vincent du Vigneaud elucidated the structure of this hormone by determining the amino acid sequence of OXT and achieving its total chemical synthesis (5–7), thereby establishing the molecular identity of the peptide responsible for these effects (work that earned him the Nobel Prize in Chemistry in 1955).

Although OXT was one of the first peptide hormones to be characterized and synthesized (8, 9), its effects were long considered to be restricted to the stimulation of uterine contractions and milk ejection during lactation. However, the detection of the oxytocin receptor (OXTR) in multiple central and peripheral tissues suggests that this neuropeptide has additional physiological functions (8, 10). Indeed, besides contractile uterine smooth muscle and mammary myoepithelial cells, OXTR expression has also been reported in skeletal muscle, although its functional role in this tissue remains poorly understood.

In this context, although Breton and colleagues were the first to directly demonstrate OXTR expression in human myoblasts in 2002 (11), earlier studies by Wakelam et al. (1987) and Nervi et al. (1995) had already provided indirect evidence suggesting the presence of functional OXTR in skeletal muscle lineage cells, as indicated by increased inositol phosphate production and enhanced differentiation in L6 myoblasts exposed to OXT, respectively (12, 13). Since then, a limited number of studies have demonstrated that OXT regulates myogenic differentiation (11), regeneration (14), and carbohydrate (15) and protein metabolism (16, 17) in skeletal muscle. Recent evidence also shows that skeletal muscle itself synthesizes and secretes OXT (18–20), indicating that this peptide may also function as a myokine (21, 22). Given that skeletal muscle accounts for approximately 40–60% of total body mass in mammals (23–26), understanding both its contribution to circulating OXT levels under different physiological and pathological conditions and the effects of this peptide on muscle structure and function may reveal novel regulatory mechanisms and potential therapeutic applications. In this context, this review compiles and discusses recent findings on the role of OXT in the regulation of skeletal muscle plasticity and metabolism, while also highlighting current knowledge gaps and future perspectives in the field.

## 2 Literature search strategy

This narrative review was developed through comprehensive literature searches conducted in PubMed, Web of Science, and Google Scholar. Given the relatively limited number of studies directly investigating oxytocin signaling in skeletal muscle, no publication date restrictions were applied.

Searches were performed using combinations of terms related to oxytocinergic and vasopressinergic signaling and skeletal muscle biology. Representative search strings included: “oxytocin AND skeletal muscle”, “oxytocin receptor AND skeletal muscle”, “OXTR AND skeletal muscle”, “oxytocin AND myogenesis”, “oxytocin AND muscle regeneration”, “oxytocin AND muscle plasticity”, “oxytocin AND proteolysis”, “oxytocin AND muscle protein metabolism”, “oxytocin AND muscle atrophy”, “oxytocin AND sarcopenia”, “oxytocin AND glucose metabolism”, “oxytocin AND insulin resistance”, “oxytocin AND diabetes”, “oxytocin AND myokine”, “vasopressin AND skeletal muscle”, “V1a receptor AND skeletal muscle”, “vasopressin AND myogenesis”, and “vasopressin AND muscle regeneration”.

Priority was given to original research articles, recent publications, and seminal studies that established key concepts in the field. Additional relevant studies were identified through manual examination of reference lists from selected articles.

## 3 Oxytocinergic signaling in skeletal muscle

OXT and vasopressin (AVP) are closely related nonapeptides synthesized and secreted by magnocellular neurons of the hypothalamic paraventricular (PVN) and supraoptic (SON) nuclei, and released into the systemic circulation via the neurohypophysis. Indeed, the two peptides differ by only two amino acid residues (8, 27). This high degree of structural similarity enables OXT to interact not only with its canonical receptor, the OXTR, but also with AVP receptors (V1a, V1b, and V2), albeit with lower affinity (27). In skeletal muscle, early functional evidence suggested the presence of AVP receptors (12, 13, 28), and it was later established that the V1a subtype is the only AVP receptor identified in this tissue (29). In this context, Wakelam et al. (1987) showed that incubation with AVP and its analogs, including OT, increased inositol phosphate production in L6 myoblast cultures (12). At that time, this effect was attributed to activation of V1-type receptors rather than V2, since no changes in intracellular cAMP levels were detected. However, the selectivity of OXT action toward V1 receptors was not directly assessed, leaving it unclear whether the observed effects were mediated exclusively by AVP receptors or also involved OXTR. Only later did Breton et al. (2002) provide molecular evidence of OXTR expression in human myoblasts, confirming the presence of functional OXTR in cells of the skeletal muscle lineage (11).

The OXTR belongs to the rhodopsin-like (class I) family of G protein–coupled receptors (GPCRs) (8–10, 30). Although its presence in skeletal muscle has been established, the intracellular signaling mechanisms triggered by its activation in this tissue remain relatively underexplored compared with other biological systems. The limited studies examining the direct effects of OXT in skeletal muscle lineage cells have shown that OXT increases inositol phosphate production in L6 myotubes (12), which appears to contribute to its myogenic effects (13). Consistently, OXT stimulation of primary myogenic progenitor cells induced rapid ERK1/2 phosphorylation, which was abolished by the MEK inhibitor U0126, demonstrating that OXTR activation engages the MAPK–ERK pathway to regulate myogenesis (14).

In addition, OXT has been shown to increase glucose uptake through a mechanism mediated by elevated intracellular Ca²^+^, leading to activation of Ca²^+^/calmodulin-dependent protein kinase kinase (CaMKK) and AMP-activated protein kinase (AMPK) in C_2_C_12_ cells (15). Interestingly, exposure of this same cell line to 17β-estradiol increased both OXTR gene and protein expression, as well as OXT levels in the incubation medium (18), supporting the notion that skeletal muscle can synthesize and secrete OXT and that the promoter regions of both genes are responsive to estrogen (E_2_) (19, 31).

**Figure 1:**
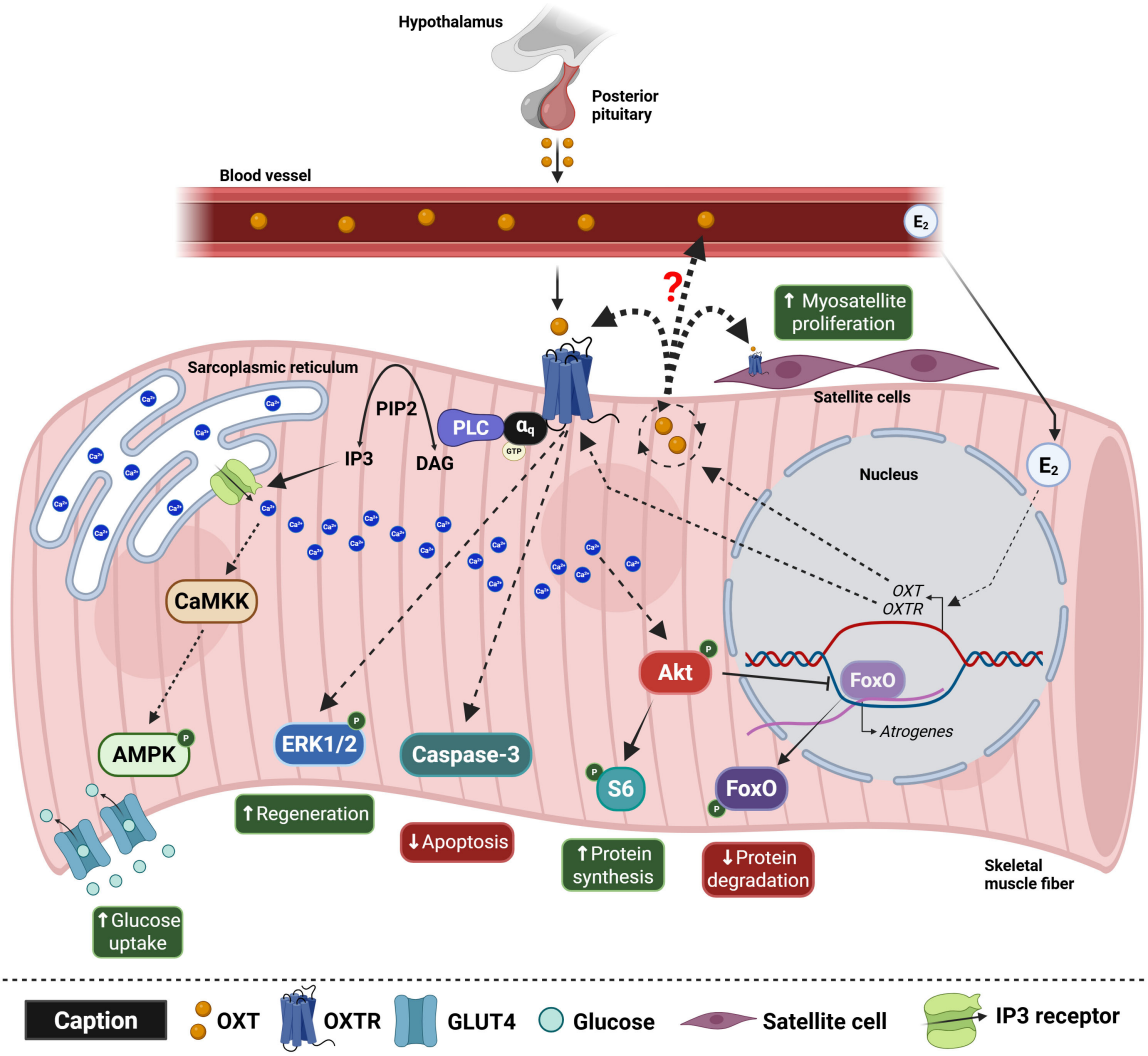
Schematic representation of oxytocinergic signaling in skeletal muscle, focusing on the main intracellular pathways activated by oxytocin (OXT) and the resulting biological effects. The image also depicts the sources of OXT, including the hypothalamus and skeletal muscle itself (acting as a myokine), and its potential autocrine, paracrine, and endocrine modes of action. The continuous arrows (→) represent steps of molecular pathways that have already been described in the literature, while the dashed arrows (⇢) represent deducted pathways or connections based on the most actual data. The (⟞) symbols represent an inhibitory step. Created in Biorender.

It is important to note that although OXTR activation is classically associated with coupling to Gαq proteins, experimental evidence indicates that this receptor can also signal through Gαi proteins (8, 10, 32). In this regard, our group recently demonstrated that OXTR activation, either by OXT or the selective nonpeptide agonist WAY-267,464, attenuates proteolysis in isolated oxidative skeletal muscles (soleus) of female rats (17). This effect was mediated by an increase in intracellular Ca²^+^, since it was abolished by 2-APB, an IP_3_ receptor antagonist that blocks Ca²^+^ release from the sarcoplasmic reticulum. In contrast, the antiproteolytic effect remained unchanged in the presence of pertussis toxin. Together, these findings indicate that, in differentiated skeletal muscle, the antiproteolytic action of OXTR activation is associated with Gαq-dependent signaling. Notably, in the same study, co-incubation with WAY-267,464 and 2-APB reduced Akt phosphorylation at Ser^473^, a modification required for its activation, and consequently for inhibition of proteasomal and autophagic proteolytic pathways. Consistent with this observation, incubation of soleus muscles with WAY-267,464 in the presence of the Akt inhibitor triciribine overcame the antiproteolytic effect induced by selective OXTR stimulation (17). Collectively, these results reinforce our previous findings (16) and point to a crosstalk between OXTR/Gαq/Ca^2+^ signaling and the classical Akt pathway, typically activated by receptor tyrosine kinases such as the insulin receptor in skeletal muscle. A summary of these findings is presented in **Figure 1**.

## 4 Regulation of skeletal muscle plasticity by OXT

Skeletal muscle has historically been classified as a non-target tissue for OXT, based on autoradiography studies using radiolabeled OXT that failed to detect tissue labeling, which was interpreted as indicative of low or absent receptor expression (32), along with a lack of direct contractile effects (33, 34). This framework contributed to delaying the recognition of muscle as a functional OXT target. However, *in vitro* studies have demonstrated that OXT exerts pro-myogenic effects by promoting myoblast fusion and myotube formation (11), while also enhancing glucose uptake (15) in muscle cells. Given that skeletal muscle is the largest tissue compartment in body mass and the primary site of insulin-stimulated glucose disposal (35), alterations in muscle mass directly impact systemic energy metabolism (25). In this context, OXT deficiency leads to early age-associated muscle loss (14), whereas obesity and insulin resistance are associated with reduced circulating OXT levels and decreased receptor expression in skeletal muscle (36–40). Moreover, both circulating OXT levels and OXTR expression in satellite cells decline with aging, leading to a diminished regenerative capacity of skeletal muscle (14). Taken together, these findings support the hypothesis that OXT regulates muscle plasticity and energy homeostasis, linking muscle mass control to systemic metabolism and emerging as a potential therapeutic target in metabolic disorders.

Supporting this view, OXT exerts multiple insulin-like actions (15, 41–43), and acts as an insulin secretagogue in pancreatic β-cells (44). Notably, reduced serum OXT levels have been reported in individuals with both type 1 and type 2 diabetes (45, 46). In parallel, postmortem analyses have revealed a marked reduction in OXT -immunoreactive neurons within the PVN of diabetic patients (47), suggesting potential involvement of the oxytocinergic system in the pathogenesis of *diabetes mellitus*. More recently, intranasal administration of 24 IU of this neuropeptide was associated with significant body mass reduction, enhanced insulin sensitivity and β-cell function (48), as well as a gain of up to 2.25 kg in muscle mass accompanied by improved physical performance in obese sarcopenic individuals (49). Whether this OXT -induced antidiabetogenic effect depends, at least in part, on a protective role in skeletal muscle mass remains an open question, warranting further investigation.

While the effects of OXT on body mass are consistent, its impact on muscle mass is more variable, ranging from maintenance to increases depending on the experimental context (50–52). Mechanistically, such heterogeneity may also arise from tissue-specific regulation of OXTR expression and peptide bioavailability. In this regard, obese Zucker (*fa/fa*) rats exhibited increased OXTR expression in adipose tissue alongside reduced protein expression in skeletal muscle (37), accompanied by lower circulating OXT levels (36). This reduction has been attributed to increased oxytocinase activity in the liver and adipose tissue, despite its lower activity in skeletal muscle (36), potentially influencing both systemic hormone levels and local availability.

Although OXT metabolism, particularly its synthesis, degradation, and regulation in skeletal muscle, remains poorly understood, evidence suggests that sex steroids play a modulatory role in this pathway. Indeed, anabolic steroid supplementation with trenbolone acetate and estradiol caused dramatic increases in OXT gene expression in skeletal muscle and circulating levels in both cattle and sheep (19, 20). Interestingly, dexamethasone-treated cattle also showed increased OXT gene expression in skeletal muscle, although elevated plasma OXT levels were observed only in animals receiving 17β-estradiol (31). These findings raise the possibility that the hypertrophic effects of sex steroids may depend on enhanced oxytocinergic signaling in skeletal muscle or even that this tissue might contribute to circulating OXT, given that it accounts for approximately 40-60% of total body mass in mammals. However, the steroid-induced increase in plasma OXT may, at least in part, result from actions on hypothalamic magnocellular neurons (53). On the other hand, evidence that 17β-estradiol increases OXT expression in C_2_C_12_ muscle cells, along with elevated peptide levels in the culture medium, indicates that skeletal muscle not only produces but also secretes OXT, supporting its classification as a myokine (18).

Physiologically, the decline in circulating sex steroid levels during aging is associated with sarcopenia and is accompanied by reduced circulating OXT levels (14). Similarly, experimental depletion of sex steroids through ovariectomy or orchiectomy induces muscle loss, a condition in which OXT treatment fails to attenuate the phenotype (54, 55), suggesting that the effects of this peptide on skeletal muscle depend on the steroidal milieu. In line with this, both circulating OXT levels and OXTR expression in muscle stem cells decline with age, and exogenous OXT treatment in aged animals restores regenerative capacity (14). The same study showed that OXT deficiency leads to reduced muscle mass and fiber size, consistent with early sarcopenia. Importantly, three-month-old OXT knockout mice do not display alterations in muscle tissue or fiber size, indicating that genetic absence of OXT does not impair muscle development and is more closely related to the progression of sarcopenia during aging (14).

Although the study by Elabd and colleagues clearly established a role for OXT in regulating muscle plasticity, particularly regeneration (14), they did not determine whether this peptide also influences protein synthesis and degradation, processes that dynamically balance to define muscle fiber size (23). Building on this premise, our group demonstrated that incubation of oxidative muscles (soleus) from female rats with the non-peptide OXTR agonist WAY-267,464 (WAY) reduced total proteolysis by approximately 25%, an effect abolished by co-incubation with the highly selective receptor antagonist L-371,257 (16). This anti-catabolic effect was mediated by attenuation of the two major proteolytic systems, the ubiquitin–proteasome and autophagy–lysosome pathways. Consistently, WAY increased phosphorylation of Akt-Ser^473^ and FoxO1-Ser^253^, while reducing the denervation-induced upregulation of the atrogenes MuRF-1 and atrogin-1, muscle-specific ubiquitin ligases whose expression rises under multiple atrophic conditions (16). Akt phosphorylation promotes its activation, leading to FoxO1 phosphorylation and nuclear exclusion, thereby reducing atrogene expression and ubiquitin–proteasome-mediated proteolysis (56). Accordingly, the anti-proteolytic effect of OXTR stimulation was proposed to involve cross-talk with insulin-mediated intracellular signaling through the Akt–FoxO pathway (16), a mechanism later confirmed by our group (17).

In the same study, we also showed that protein synthesis, assessed by puromycin incorporation, was unchanged in muscles under basal conditions incubated with WAY, but was increased in oxidative muscles from animals treated with OXT (intraperitoneally), indicating that anabolic effects on protein metabolism occur indirectly. Similarly, the treatment was accompanied by increased phosphorylation of ribosomal protein S6 at Ser^235/236^, a modification involved in the regulation of protein synthesis through the promotion of mRNA translation (16). Although insulinemia was not measured, the hypertrophic effect observed in soleus muscle may have resulted from enhanced insulin secretion induced by OXT administration (57), given that insulin is a key anabolic hormone in skeletal muscle (58). In parallel, a contribution of the sympathetic nervous system (SNS) cannot be excluded, since it is well known to exert anabolic and anti-catabolic effects on muscle protein metabolism (24, 26, 59).

Indeed, OXT has been shown to increase the neural activity of the SNS (60) and to exert trophic effects on the adrenal medulla, enhancing catecholamine secretion (61). This is supported by studies with OXT ^−/−^ animals, which display reduced adrenaline secretion and lower energy expenditure (38), possibly associated with decreased muscle mass (39). This hypothesis is further supported by the observation that the hypertrophic effect of OXT occurs primarily in oxidative muscles (16), which have a higher density of β2-adrenergic receptors compared to glycolytic muscles (62), and regulate protein metabolism via the cAMP/PKA pathway (63–65). Additionally, cold exposure increases SNS activity (26), and is accompanied by elevated OXTR expression in oxidative muscles (66). Given that the OXTR gene contains cAMP-responsive elements (67), sympathetic activation may modulate oxytocinergic signaling in muscles. Moreover, it has recently been shown that tyrosine hydroxylase-positive sympathetic neurons innervating white adipose tissue co-express OXT, which potentiates β-adrenergic lipolytic responses (68). Whether this OXT -positive sympathetic innervation also directly targets skeletal muscle remains unknown and warrants further investigation.

Additionally, in an ischemia–reperfusion model, OXT attenuated apoptosis and reduced caspase-mediated proteolytic activity, thereby preserving muscle tissue (69). In older individuals, in whom inflammaging exacerbated exercise-induced inflammation and muscle fibrosis, OXT combined with a TGF-β inhibitor (Alk5) attenuated these effects, restored more “youthful” proteomic profiles, and normalized key metabolic pathways such as Akt signaling, promoting regeneration and tissue maintenance (70). OXT was also shown to reverse cancer cachexia-associated alterations, restoring myogenic potential, muscle mass, and strength, while reducing the expression of atrogenes such as MuRF1 and atrogin-1, further supporting its role in modulating muscle protein metabolism (71). Taken together, these findings position OXT as a promising candidate for interventions aimed at preserving muscle mass, although its clinical application still depends on studies defining safety, dosing strategies, and potential interindividual variability related to OXTR.

A summary of the current evidence regarding OXT signaling and its role in skeletal muscle physiology, metabolism, and plasticity is presented in **Table 1**.

**Table 1:** Experimental and clinical evidence supporting oxytocin actions in skeletal muscle.

Main finding	Experimental approach	Reference
Increased IP_3_ production in myoblasts following OXT stimulation	*in vitro*	(12)
Identification of OXTR expression in human myoblasts Increased myoblast fusion and myotube formation	*in vitro* *in vitro*	(11)
Increased glucose uptake via CaMKK/AMPK signaling in C_2_C_12_ cells	*in vitro*	(15)
Reduced apoptosis and caspase-mediated proteolytic activity following OXT treatment	*in vivo*	(69)
OXT-induced ERK1/2 phosphorylation in myogenic progenitor cells Reduced skeletal muscle regenerative capacity associated with decreased OXT levels OXT deficiency is associated with reduced muscle mass and fiber size (sarcopenic phenotype)	*in vitro* *in vivo in vivo*	(14)
Estradiol-induced OXT expression in C_2_C_12_ cells, supporting a myokine role for OXT	*in vitro*	(18)
Reduced ubiquitin–proteasome- and autophagy-mediated proteolysis in soleus muscles following selective OXTR activation Increased protein synthesis in soleus muscle following OXT treatment *in vivo*, but not after direct ex vivo exposure	*ex vivo* *in vivo/ex vivo*	(16)
Increased muscle mass and improved physical performance in obese individuals treated with OXT	*in vivo*	(49)
OXTR activation engages a Gαq/Ca^2+^/Akt signaling pathway in soleus skeletal muscle	*ex vivo*	(17)
Reduced fibrosis and inflammation in older individuals following OXT treatment	*in vivo*	(70)
Restoration of myogenic potential, muscle mass, and muscle strength in cancer cachexia	*in vivo*	(71)

## 5 Perspectives and conclusions

Across multiple physiological and pathological contexts, changes in circulating OXT are consistently associated with alterations in muscle mass. For example, physical exercise promotes muscle hypertrophy (72) and is accompanied by increased circulating OXT (73), whereas diabetes (45, 46) and aging (14) are characterized by muscle loss alongside reduced OXT levels. Similarly, anabolic steroid administration enhances muscle mass while increasing both circulating and muscle-derived OXT (18–20). Despite these consistent associations, the causal role and underlying mechanisms by which OXT regulates muscle mass and function in these conditions remain poorly understood.

Dehydration and lactation represent additional physiological states of increased OXT secretion, driven by its roles in natriuresis (53) and milk ejection (4, 8, 40). During lactation, substantial loss of body mass occurs while skeletal muscle appears relatively preserved, although this remains incompletely established (74, 75). In contrast, our group demonstrated that dehydration induced muscle loss accompanied by increased activity of hypothalamic OXT neurons (25). In both conditions, the role of OXT in regulating skeletal muscle mass remains unclear; however, these observations raise the possibility that OXT may attenuate muscle wasting during dehydration and potentially preserve muscle mass during lactation, contributing to tissue protection under conditions of high metabolic demand.

Skeletal muscle contraction depends on motor cholinergic input, such as that required during exercise or cold exposure (59, 76). However, during cold exposure, an increase in muscle proteolysis was demonstrated, which was attenuated by sympathetic activity (26). Interestingly, other studies have shown that this sympathetic activation during cold exposure is accompanied by increased OXTR expression in muscle (77). Furthermore, the sciatic nerve, typically motor, contains OXTR -positive neurons (78) as well as sympathetic innervation (59). Given that sympathetic input can elevate cAMP (64) and that OXTR promoter regions are responsive to cAMP (67), along with evidence that sympathetic terminals can release OXT (though it is unknown whether this occurs in skeletal muscle) (68), these observations raise the possibility of local cross-talk between sympathetic and OXT signaling. Such interactions could help preserve muscle mass during cholinergic-driven contractions, which otherwise promote proteolysis, as occurs during exercise or shivering in response to cold. However, the extent to which sympathetic–OXT cross-talk contributes to muscle preservation under these conditions remains unknown and represents an important open question for future investigation.

Besides these contexts, key mechanistic questions also remain, including how OXT synthesis, degradation, and secretion are regulated within skeletal muscle, whether muscle-derived OXT acts in an autocrine, paracrine, or endocrine manner; and whether sympathetic neurons locally release OXT within this tissue. Notably, the lack of a well-validated antibody for OXTR continues to hinder precise characterization of its localization and regulation in skeletal muscle (79).

Despite these uncertainties, compelling evidence indicates that OXT exerts protective effects on skeletal muscle, positioning it as a key integrator of muscle mass regulation and systemic metabolism with relevant therapeutic potential.
